# Integrated alternative approaches to select feed-efficient rainbow trout families to enhance the plant protein utilization

**DOI:** 10.1038/s41598-024-54218-2

**Published:** 2024-02-16

**Authors:** Kimia Kajbaf, Ken Overturf, Vikas Kumar

**Affiliations:** 1 Department of Animal, Veterinary and Food Sciences, Aquaculture Research Institute, University of Idaho, Moscow, 83844 USA; 2 ARS-USDA, Hagerman Fish Culture Experiment Station, 3059-F National Fish Hatchery Road, Hagerman, 83332 USA

**Keywords:** Biological techniques, Physiology

## Abstract

Improving feed utilization efficiency is a challenge in aquaculture. Therefore, we developed an indirect benchmark to use in selecting trout for improved efficiency of feed utilization on plant protein (soy)-based diets, with the long-term goal of reducing the cost of commercial trout production. We used a four-part integrative approach to identify feed efficient individuals among 1595 fish coming from 12 genetically selected families by establishing the phenotypic relationship between feed conversion ratio (FCR) and body weight variations using compensatory feeding regimes. Additionally, we examined the nutritional composition of fish filet for each efficiency phenotype during the compensatory regimen. Our findings showed that the fish with the lowest weight loss during a feed deprivation period and the highest weight gain during the refeeding period (FD−/RF +) demonstrated the lowest FCR (FCR = 0.99) and consisted of individuals from several lines. This finding confirms the possibility of improving feed efficiency in mixed lines. Although feeding period has an effect on nutritional composition of fillet, such selection criteria did not show an effect on groups. Overall, successful selection for the improvement of feed efficiency will have a broad application to commercial fish selective breeding programs, leading to increased aquaculture sustainability in the long run.

Aquaculture is the fastest growing food-producing sector, and it comprises half of the total fish supply sector in both the U.S. and the world. In recent years, the production of carnivorous fish such as rainbow trout in the aquaculture industry has experienced a massive expansion worldwide^1^. This rapid development has been mainly expedited using fishmeal as the major protein in most feeds, which is the most important protein ingredient owing to a high nutritional and palatability value. Replacement of fishmeal as the major protein source in feeds is critical for continued growth of the aquaculture industry as well as development of sustainable aquaculture^2^. Despite the presence of all essential nutrients, including amino acids, in the diet above the required levels, various studies have revealed that fish fed low fishmeal-high plant protein feeds experience suboptimal growth performance and reduced protein retention efficiency^3,4^. Recent commercial attempts to rear high-value, carnivorous marine species, such as cobia *Rachycentron canadum*, barramundi *Lates calcarifer* and amberjack *Seriola dumerili*, using low-fishmeal, high plant protein diets have failed^5,6^. Growth of many marine fishes is reduced when soy proteins, a major sustainable protein replacement, replace more than 20% of fishmeal protein.

Feed, which covers more than 50% of production costs, is a major concern when considering the potential negative impacts of aquaculture on the environment. Therefore, improving feed efficiency (FE) will result in reduced cost and environmental impacts of aquaculture. These issues are particularly impactful on trout (*Oncorhynchus mykiss*) culture which is ranked second in freshwater fish raised in the US^1^. Several production related traits are known to be genetically variable in fish^7^. Currently, the utilization of domesticated and genetically enhanced farm animals and plants is widespread. However, in the case of fish and shellfish aquaculture, breeding programs aimed at genetic improvement have been infrequently implemented and most of them are focused on selection based on disease resistance. In fact, only a small proportion, ranging from 1 to 2%, of aquaculture production relies on genetically improved stocks^7^ Feed utilization efficiency has shown genetic variability in various species of fish^8,9^. In general, one of the commonly used measures of feed efficiency is feed conversion ratio (FCR), the ratio of feed consumed to unit growth. Based on index theory calculations, previous studies have shown that direct selection to improve FCR, which is simultaneous selection for weight gain and against feed intake, increased the expected genetic response in FCR by 1.50-fold in rainbow trout. This increase is in comparison to solely selecting for growth^10,11^. Since recent experiments on fish have revealed that significant genetic variability exists for FE in fish, improvements of this trait can be expected with selective breeding^12,13^. However, this improvement can be more complicated because fish are reared in groups and individual feed intake is hard to be recorded directly. Also, because the domestication process of aquaculture species is more recent, reference models or inbred lines are yet to be developed^14^.

Although selection for growth is often used to indirectly improve feed utilization efficiency, it does not consider the importance of baseline energy required for maintenance and body weight gain (BWG)^12,14,15^. For that particular reason, BWG is not an accurate selection criterion in fish. While some experiments showed a positive genetic correlation between growth and feed efficiency^12,16,17^, some others found no improved feed efficiency through selection for growth^18,19^. Different selection arrangements and experimental conditions such as strain, age and water temperature may explain the variations between studies. In fish, minimum requirements for maintenance are roughly calculated by measuring energy loss at zero intake^21^. Since fish have low maintenance requirements compared to mammals, they can tolerate long periods of feed deprivation, and are able to improve their performance in terms of feed intake and efficiency to recover once they are re-fed^13,20,22,23^. Hence, improvement of FCR in fish selective breeding programs mostly rely on traits such as growth rate which in turn improves feed retention ratio and FCR^24,25^.

In rainbow trout, there is a high genetic correlation, ranging from 0.63 to 0.99, between FCR and growth rate^26^. Therefore, selective breeding of fish species offers a substantial opportunity for increasing feed efficiency, production and, ultimately, profitability in aquaculture industries. Growth and feed utilization traits are of economic importance to the aquaculture industries. Growth and feed utilization traits are of economic importance to the aquaculture industry. For eight generations (16 years), University of Idaho in collaboration with US Department of Agriculture’s Agricultural Research Service has developed a strain of rainbow trout (UI ARS-CX strain) using selective breeding that exhibits high growth rates when fed an all-plant protein feed. The UI ARS-CX strain is a unique model to identify genetic and physiological parameters associated with sustainable plant protein utilization in fish. They grow rapidly when fed all plant-protein based diets, unlike unselected trout that exhibited 10–15% lower growth than selected trout^27^.

The objective is to sort individuals into groups based on their weight loss and weight gain variations among individuals/families during the consecutive feed deprivation (FD) and re-feeding (RF) periods. We assume that the observed variation can reflect feed utilization efficiency in the population. As weight measurement is an easy and practical method in the farm setting and does not require any special equipment, weight loss and gain during FD and RF respectively, could be a relevant indirect criterion to select the metabolically efficient animals that use less feed to grow. To test such assumption, we set up a protocol to assess correlation between FCR and body weight variations during FD and RF. Since selection according to weight variations can translate to body composition variations as observed in previous studies^28^, we then analyzed the muscle quality.

## Material and methods

**Figure 1:**
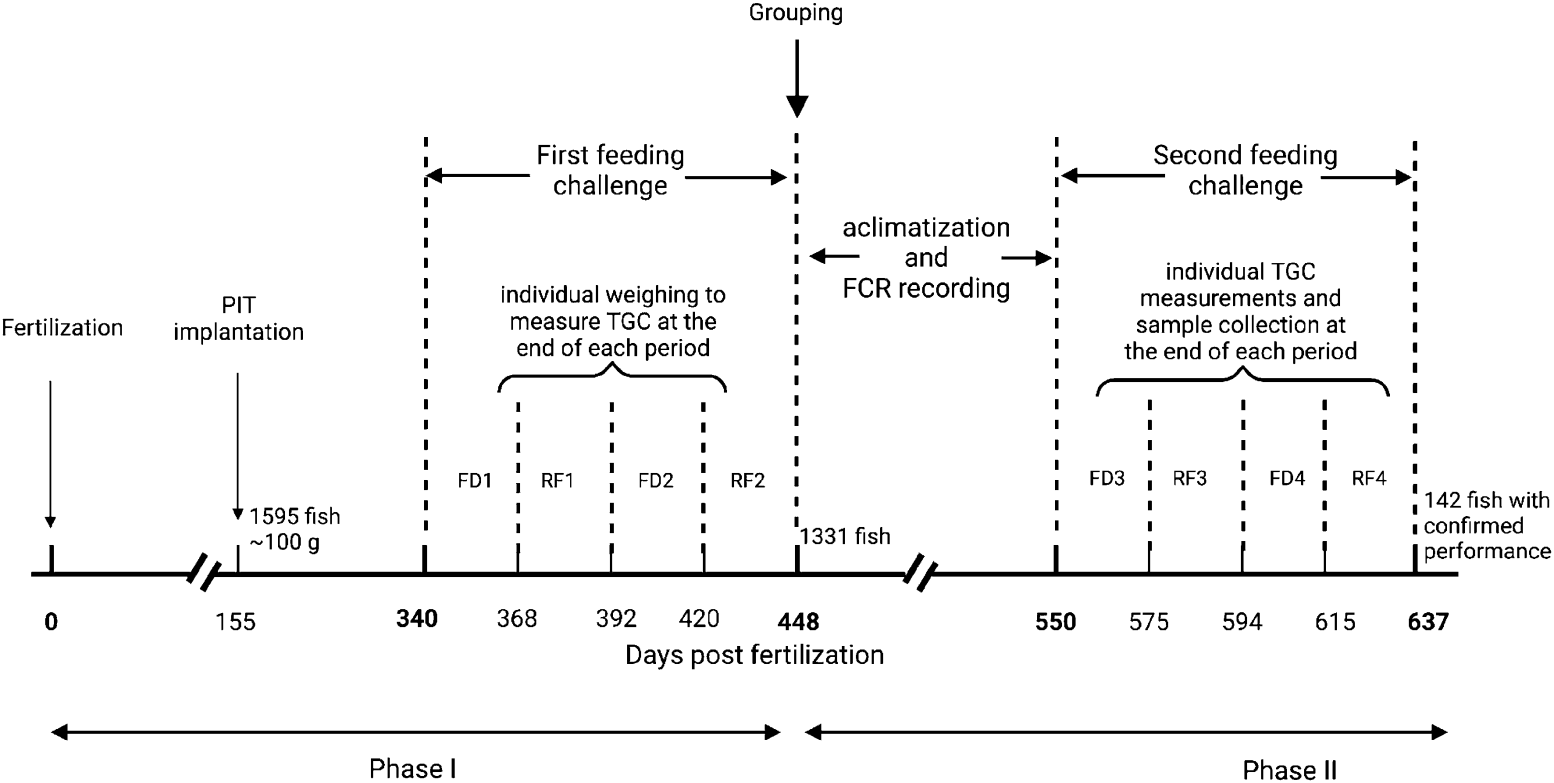
Experimental outline schematic. *FD* Feed deprivation; *RF* Re-feeding; *TGC* Thermal growth coefficient. Illustration Created with BioRender.com.

General approach and time frame of the study are shown in Fig. 1. We used twelve families from UI ARS-CX selected brood stock produced at Hagerman Fish Culture Experiment Station (HFCES) of University of Idaho. All the fish were raised in a common environment as early as possible. This study consists of 2 major phases and 4 steps; *Step 1:* Based on the previous family tree the eggs were fertilized and the 12 families were kept in separate tanks until the fish attained an average weight of 100 g (155 days post fertilization). *Step 2:* A total of 1595 fish from 12 families were randomly distributed among 4 tanks. Fish performance (body weight) during two consecutive series of FD and RF were recorded and they were classified into 4 triplicated groups according to weight gain ( +) or loss (-) relative to the population mean. At the end of this step fish were separated into 4 triplicate groups FD − /RF − , FD + /RF + , FD − / RF + and FD + /RF − ., *Step 3:* After one month of acclimatization, FCR for all four groups was calculated during a 4 months period. *Step 4:* Fish performance was re-evaluated based on the same FD and RF criteria in step 2. Muscle composition and fatty acid profile of each group were evaluated by sampling during the FD and RF periods of the second phase.

### Ethical permits/approval

All research involving experiments on fish (Rainbow trout) was approved by a licensed ethics committee of University of Idaho, USA. The experimental protocols together with fish handling and sampling were approved by the Institutional Animal Care and Use Committee (IACUC) of the University of Idaho (IACUC-2018-44), Moscow, ID, USA. All methods were performed in accordance with the Institutional Animal Care and Use Committee (IACUC) of the University of Idaho (IACUC-2018-44), Moscow, ID, USA.

### Fish production

The 12 full-sib families used in this study were produced through crossing in 1:1 ratio by crossing a single female's eggs with milt from a single male. These 12 families were a part of 200 nucleus families produced during the spawning cycle (in continuation of the work from Overturf et al.^27^) of 2017. Fertilized eggs were reared in heath trays and viable eggs then transferred to 140 L fiberglass tanks. Fish were reared in outdoor tanks in a flow-through spring water system with constant temperature of 14 ℃ throughout the study. Standard rearing protocols were used throughout the experiment (except the FD periods) and the fish were fed ad libitum using a plant protein and fish oil diet with 41% crude protein and 20% lipid. Diet formulation can be found in Table 1. Diet at the larval stage is similar to Overturf et al.^27^.

**Table 1:** Experimental diet with all plant-based protein.

Ingredient name	g/100 g
Soybean Meal, Solvent extracted	25
Wheat flour	13.3
Wheat gluten meal	2.24
Fish oil, whitting trimmings oil	17
Soybean protein concentrate, profine	23
Vitamin Premix, ARS702	1
Trace min premix ARS 1520	0.1
Stay-C	0.2
Lecithin	2
Taurine	0.5
Astaxanthin	0.08
Corn protein concentrate, E75	10.23
Dicalcium Phosphate	2.85
Lysin-HCL	1.67
DL-Methionine	0.6
Threonine	0.23
Proximate composition (% as-is basis)	
Moisture	6.90
Protein	41.23
Lipid	20.52
Ash	5.36

#### Experimental phase I

##### First feeding challenge

Group composition based on performance during compensatory feeding.

At 155 days post fertilization (pf), when fish reached an average weight of 100 ± 12 g, 133 fish from each family were randomly selected and Passive Integrated Transponders (PIT) were implanted. Then the fish from families were randomly distributed in 4 tanks (1500 L) provided with flow through spring water. At 340 pf individual weights were recorded for the initial weight before first compensatory feeding challenge consisting of two consecutive series of FD and RF. FD1 and FD2 at 340–368 pf and 391–420 pf and RF1 and RF2 at 369–392 pf and 421–448 pf respectively. At the end of each FD or RF period individual weights were recorded. Feeding was stopped 24 h prior to weighing for the initial and RF timepoints. Fish were anesthetized (40 mg/l MS-222 (tricaine methanosulfonate), buffered to pH 7.0), PIT tags were scanned, and weights were recorded to the nearest 0.1 g. A day after measurements, feeding was resumed only if the following period was RF.

Thermal growth coefficient (TGC), described as a stable measure of growth which is not affected by temperature, body weight or growth intervals was used to measure growth rate during the feeding challenges^29^. TGC can be calculated as:

$$w_{t}^{0} .33 = w_{i}^{0} .33 + (T/1000)t$$

where w_i_ and w_t_ are the body weight at the beginning and end of the considered period of time in days. T is average water temperature during the measured period in °C and t is the period of measurement in days. In this study growth rate, expressed in form of TGC, is used as a phenotypic criterion to segregate the fish.

##### Grouping the fish

Using a multiple regression, the residual values were computed on each individual during the feed deprivation and re-feeding periods and the grouping of fish was performed using the customized method detailed in Grima et al.^13^. In short, values of each individual were averaged for deprivation and refeeding trials and then the average values (deprivation vs. refeeding) were adjusted for initial differences in body weight of fish at pit-tagging (155 pf). The residuals were then standardized and scaled at mean of 0 and standard deviation of 10. The individuals which ended-up above the mean (i.e., > 0) value during deprivation were annotated as FD + with negative values of FD_corr_ and the fish which showed below mean (i.e., < 0) were called FD- with positive values of FD_corr_ (Table 2). Similarly, the individuals were annotated in refeeding with annotations of RF + and RF− and hence each individual could be called as FD + /RF + , FD + /RF−, FD−/RF + , or FD−/RF− which generated four groups of individuals. The individuals near the mean of zero were not grouped in any category to possibly avoid addition of false positive individuals. Hence, an arbitrary low value of 1.5 distance from the mean was applied to discard all the individuals concentrated near the mean. The filtration process retained 1332 individuals with FD−/RF− = 397, FD−/RF +  = 258, FD + /RF− = 368, and FD + /RF +  = 309.

**Table 2:** Means ± SE of the thermal growth coefficient (TGC) in rainbow trout during the two feeding challenges. TGC recorded for a basic growth period (BG), followed by two periods of four weeks feed deprivation (FD1 and FD2) alternated with two periods of four weeks ad libitum re-feeding (RF1 and RF2), TGC recorded at the Inter-challenge period (IC) followed by two periods of four weeks feed deprivation (FD3 and FD4) alternated with two periods of four weeks ad libitum re-feeding (RF3 and RF4), for the four groups of fish with variable performance in terms of weight loss during feed deprivation (FD − /FD +) and weight gain during re-feeding (RF − /RF +). n gives the number of fish in each group.

	FD−/RF +	FD−/RF−	FD + /RF +	FD + /RF−	*P*−value
First challenge					
n	253	310	399	369	
TGC-BG	0.078 ± 0.004	0.077 ± 0.005	0.079 ± 0.005	0.08 ± 0.005	0.17
TGC-FD1	0.088 ± 0.092	0.069 ± 0.105	−0.1 ± 0.099	−0.127 ± 0.075	5.10E−211 ***
TGC-RF1	−0.022 ± 0.025	−0.086 ± 0.112	−0.024 ± 0.052	−0.065 ± 0.088	3.05E−32 ***
TGC-FD2	0.106 ± 0.039	0.148 ± 0.1	0.037 ± 0.143	0.111 ± 0.035	4.70E−51 ***
TGC-RF2	−0.016 ± 0.039	−0.054 ± 0.039	0.033 ± 0.14	−0.053 ± 0.08	3.29E−47 ***
FDcorr	2.26	0.15	−2.15	−0.79	< 2.2e−16 ***
RFcorr	1.45	0.33	−0.57	−0.93	< 2.2e−16 ***

Second challenge					
n	193	190	150	187	
TGC-IC	0.35 ± 0.047	0.36 ± 0.047	0.38 ± 0.056	0.38 ± 0.057	0.297
TGC-FD3	−0.063 ± 0.021	−0.073 ± 0.088	−0.064 ± 0.018	−0.068 ± 0.013	0.193
TGC-RF3	0.133 ± 0.274	0.133 ± 0.111	0.142 ± 0.048	0.135 ± 0.125	0.957
TGC-FD4	−0.047 ± 0.259	−0.043 ± 0.03	−0.058 ± 0.109	−0.051 ± 0.112	0.819
TGC-RF4	0.195 ± 0.149	0.228 ± 0.026	0.214 ± 0.115	0.226 ± 0.039	0.647
FDcorr	2.03	1.90	−1.54	−1.14	< < 2e−16 ***
RFcorr	1.96	2.27	−2.77	−2.32	< 2.2e−16 ***

***The p value less than 0.05 are significantly different among the four groups.

#### Experimental phase II

##### Recording FCR

After grouping, 258 fish (14% of the initial population) from each group were randomly selected to move on to the second phase. This number for each group was dictated by the number of fish in the group which has the fewest individuals. The fish from each of the 4 groups, constituted after the first feeding challenge, were distributed equally among 3 tanks (total of 12 tanks) and spent a month of acclimatization before the FCR recording period began. From day 478 pf to 550 pf the fish were fed ad libitum 2 times a day (6 days a week) and fish were weighed once at each end of this period to calculate FCR:

$$\text {FCR} = \text {The total weight of the feed intake (g)}/ \text {Total weight gain by fish (g)}$$

##### Second feeding challenge: re-evaluation of groups

A second feeding challenge similar to the first one was performed with initial weights recorded at day 550 pf followed by two consecutive series of FD and RF. (FD3 and FD4 at 551–557 pf and 595–615 pf, respectively and RF3 and RF4 at 576-594pf and 616–637 pf. Individual fish weights were recorded as described before at the end of each period. Also, 3 fish per tank (total of 9 fish per group) were randomly collected and euthanized (300 mg/l MS-222) to collect fillet samples for fillet composition and fatty acid profile measurements.

##### Proximate composition and fatty acid composition of fish fillet

At the end of each FD and RF period during the second challenge, 3 fish per tank (9 fish per group) were euthanized using an overdose of (MS-222; Western Chemical Inc, Ferndale, WA) (300 mg L^−1^) and after recording the PIT tag ID fillets from both sides of each fish was packed and kept frozen in –20 °C for proximate analysis and determination of fillet fatty acid profile. Proximate composition analysis (%) started with pureeing and homogenizing feed and fillet via an industrial food processor, then drying at 105 °C for 24 h (to determine moisture level), and grounding with mortar and pestle for further analysis. LECO FP-428 nitrogen analyzer (LECO Instruments, St. Joseph, MI) was used to determine crude protein by measuring the total nitrogen and multiplying by 6.25. Petroleum ether was used as an extracting solvent to analyze crude lipid per ANKOM XT15 extraction apparatus (ANKOM Technology, Macedon, NY) manufacturer’s instructions. Dried and powdered tissue was incinerated at 550 °C to measure ash.

The fatty acid profiles of rainbow trout homogenized filet samples were determined according to a method modified from AOAC method 991.39. About 0.5 g of homogenized and mixed fillet samples were dried at 50 °C under a nitrogen stream for 5 h. After drying, 2.0 ml of 0.5N KOH was added and the samples saponified for 60 min at 70 °C. After cooling, the free fatty acids were methylated by adding 2.0 ml of 14% Boron Trifluoride (BF3) in methanol and incubated at 70 °C for 60 min. The samples were cooled, 2.0 ml of hexane was then added, the samples repeatedly inverted for 1 min, and 2.0 ml of saturated sodium chloride was added. The samples were again repeatedly inverted for 1 min and then centrifuged for 5 min at 2000 g. 100 µl of the clarified hexane extract was added to 900ul hexane (1:10) in autosampler vials for GC/MS analysis. The Thermo 1310 GC was operated in the split injection mode with a helium flow rate of 2 ml/min and a split ratio of 1:3. The injection port was maintained at 250 °C and the transfer line at 260 °C. The initial column temperature of 100 °C was held for 0.2 min, then ramped at 30 °C/minute to 150 °C, then at 2 °C/minute to 180 °C, and then at 15ºC/minute to 240 °C where it was held for 1 min. Sample injections of 1 µl provided adequate response in the ISQ mass spectrometer. The MS was operated in the scan mode from 50–450 m/z for the period of 4.0 to 21.7 min post-injection. This program allowed for analysis of fatty acid methyl esters (FAMEs) of C14:0 to C24:1 with a run time of 22 min. The column used was a Zebron FAME, 30 m long, 0.25 mm id, with a film thickness of 0.20 µm. Muscle chemical composition and the fatty acid profile for each group is reported as the mean of 9 individuals sampled from that group at each time point.

#### Statistical analysis

To report body weight gain, thermal growth coefficient, chemical composition and fatty acid profile, group means at each time point were used as units of observation for statistical analysis. Using Python 3.7.1 parameters were analyzed and tested for normality and homogeneity of variance by using the residuals and normal Q-Q plots and Bartlett’s Test. A one-way ANOVA was used to report significant differences among groups in terms of body weight gain and thermal growth coefficient at each period and a two-way factorial ANOVA with an interaction effect was performed to find significant differences in proximate composition and fatty acid profile of fillet.

## Results

### Phase 1

**Figure 2:**
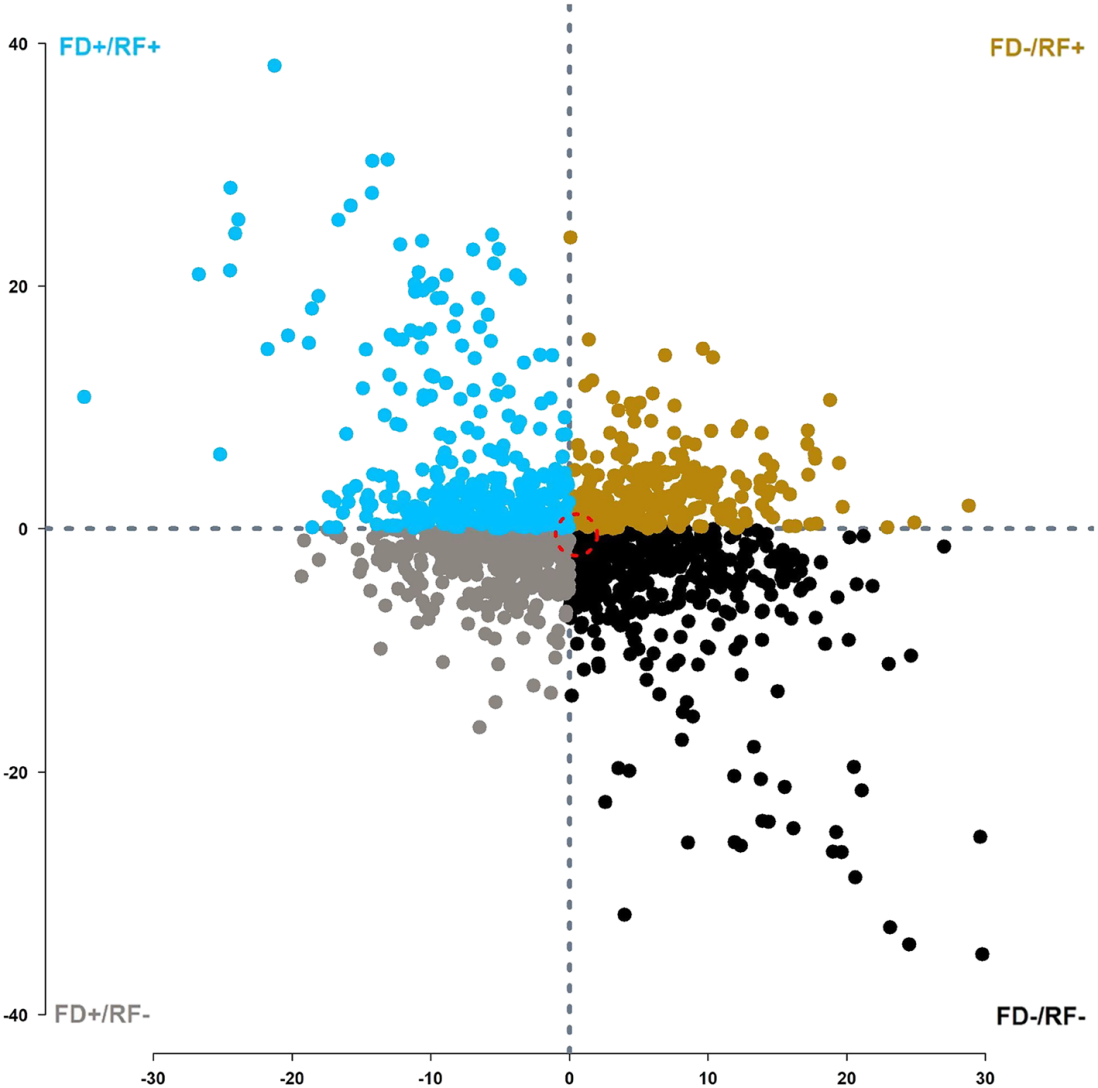
Body weight performance during the first feeding challenge 340–448 pf. FD- and FD + refer to fish with small and high weight loss during a feed deprivation period, RF- and RF + refer to small and high weight gain during a refeeding time. Individuals within the limits of the dotted line (at the center) were not classified with this criterion. Performance is shown as residual values computed through multiple linear regression of TGC.

**Figure 3:**
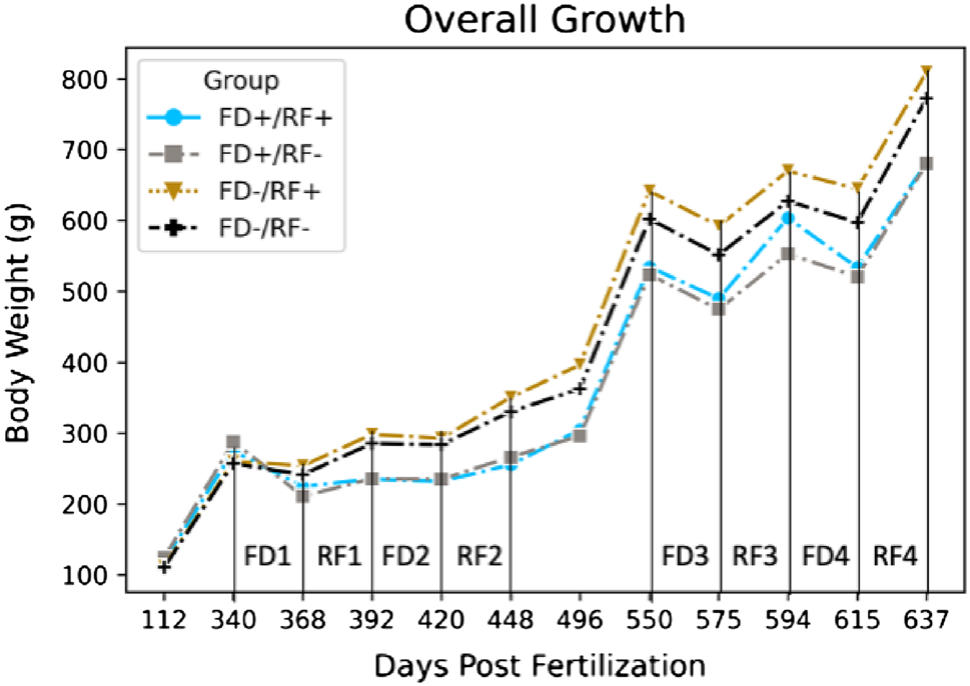
Growth over the entire experimental period.

Rainbow trout tolerated the long periods of feed deprivation and the survival rate over the two phases of the experiment was at the sustainable rate of > 98%. Similar rates had been observed in previous research and was expected. Although throughout the 526 days of this trial an overall increase in body weight was observed (Fig. 3 and Table 3), large growth variation was detected among the individuals during the first phase of the experiment (Fig. 2 and Table 2). Such variation in performance allowed for sorting the fish based on the weight gain and loss criteria explained earlier (Fig. 2 and Table 2). After, the fish were grouped based on weight loss during FD and weight gain during RF, the best performing group (FD−/RF +) showed an overall higher weight throughout the whole experiment (Fig. 3). Although their TGC during the basic growth period was statistically similar (*P* = 0.17), TGC was always significantly different among groups during the first feeding challenge (*P* < 0.001). While an overall increasing trend was observed in daily BWG per fish (Fig. 3), significant differences among the four groups during the two FD and RF periods of the first feeding challenge were detected. Interestingly, Fish BWG during the second FD period of both challenges (FD2 and FD4) was not observed to be significantly different (Table 2).

**Table 3:** Body weight gain (BWG, g fish^−1^ day^−1^) variations existed in the population throughout the study. FD = Feed deprivation and RF = Re feeding.

Feeding challenge period	FD−/RF +	FD−/RF−	FD + /RF +	FD + /RF−	*p*-Value
First challenge					
BWG					
FD1	−0.22 ± 0.97	−0.56 ± 1.27	−1.79 ± 1.39	−2.74 ± 1.50	2.45E−75***
RF1	1.84 ± 2.04	1.83 ± 1.64	0.43 ± 0.77	1.01 ± 0.51	4.47E−23***
FD2	−0.19 ± 1.33	−0.06 ± 1.05	−0.11 ± 1.12	−0.01 ± 0.82	0.45
RF2	2.06 ± 2.02	1.66 ± 1.58	0.83 ± 2.02	1.11 ± 1.04	1.66E−11***
Second challenge					
BWG					
FD3	−1.61 ± 0.58	−1.7 ± 1.13	−1.46 ± 0.5	−1.45 ± 0.33	0.001442**
RF3	3.99 ± 2.26	4.15 ± 2.05	4.28 ± 1.75	3.91 ± 2.62	0.0422321*
FD4	−1.16 ± 2.09	−1.41 ± 1.05	−1.6 ± 1.85	−1.57 ± 2.23	0.093655963
RF4	7.44 ± 3.48	8.1 ± 1.41	6.94 ± 2.67	7.09 ± 1.77	4.13E−05***

*, **, ***The p value less than 0.05 are significantly different among the four groups.

### Phase 2

#### Growth

**Figure 4:**
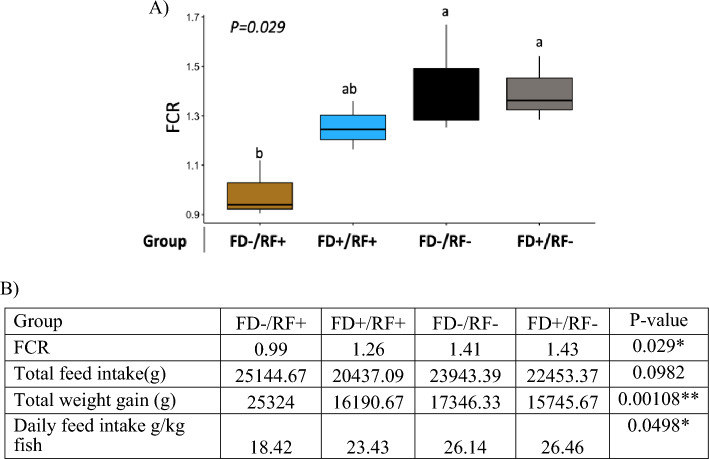
(**A**) Following the first phase of experiment, FCR was recorded between days 478 and 550 dpf. (**B**) Growth and feed intake parameters for the four groups.

The second phase started with a month of acclimatization so the fish can recover from the feeding challenge and FCR was measured afterwards during a four-month ad-libitum feeding. FCR measurements revealed that the groups with lower body weight gain during a refeeding episode (RF-) had the significantly higher FCR (Fig. 4). This may indicate that higher growth rate is due to more successful weight gain during RF rather than lower weigh loss during FD. While the FCR value among the 4 groups was found to be significantly different (*P* = 0.029), the FD-/RF + group with lower body weight loss during feed deprivation and higher weight gain during refeeding period showed the lowest FCR among all (FCR = 0.99) (Fig. 4). Additionally, the FD-/RF + group with the lowest FCR shows the lowest daily feed intake value (Fig. 4). Like the first phase, the best (FD−/RF +) and worst (FD + /RF−) preforming groups have always had the highest and lowest weight among the four groups respectively (Fig. 3).

#### Grouping

**Figure 5:**
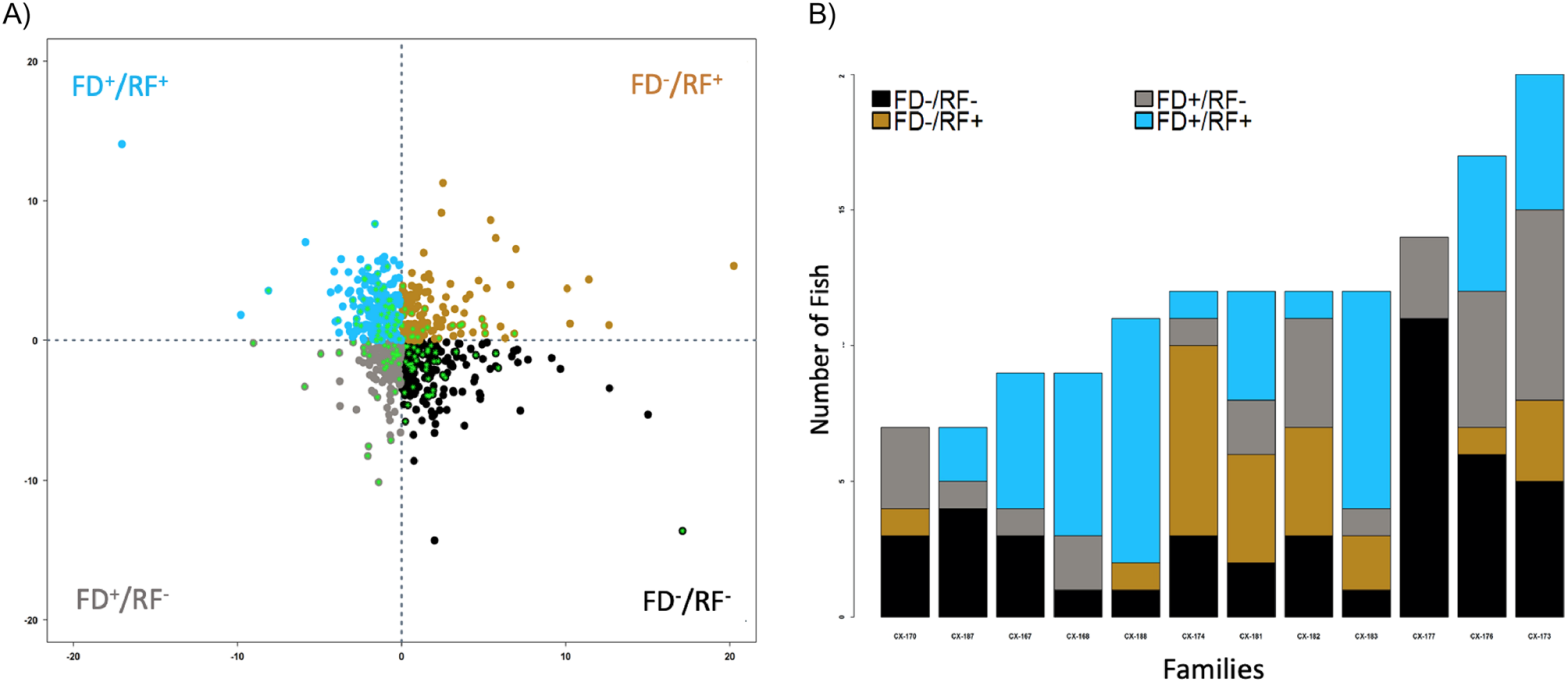
(**A**) body weight performance during the second feeding challenge 550–637 dpf. FD- and FD + refer to fish with small and high weight loss during a feed deprivation period, RF- and RF + refer to small and high weight gain during a refeeding time. Performance is shown as residual values computed through multiple linear regression of TGC. Fluorescent green asterisks represent the individuals with similar performance during the two feeding challenges. (**B**) Distribution of individuals with similar performance in two challenges among families.

During the second phase of this study, once the fish were re-evaluated based on the same criteria as the first phase (Fig. 5a), 142 fish (14.03% of the population in the second challenge) demonstrated the same performance and fell in the same groups as last time (shown in fluorescent green in Fig. 5a). These fish are unequally distributed among the 12 families (Fig. 5b) with FD−/RF + 16.2%, FD + /RF− 21.13%, FD−/RF− 30.3% and FD + /RF + 32.4% of the population. During the second challenge, in contrast to the first feeding challenge, no TGC was determined significantly different among the 4 groups in any of the FD or RF periods. The mean TGC values were always negative during FD and positive during RF for all groups (Table 2). Daily body weight gains during the second feeding challenge were significant in FD3, RF3 and RF4, but not FD4 (Table 3). During the course of the study, weight loss during FD and weight gain during RF periods were constantly observed for all groups (Table3 and Fig. 3).

#### Nutritional composition

**Figure 6:**
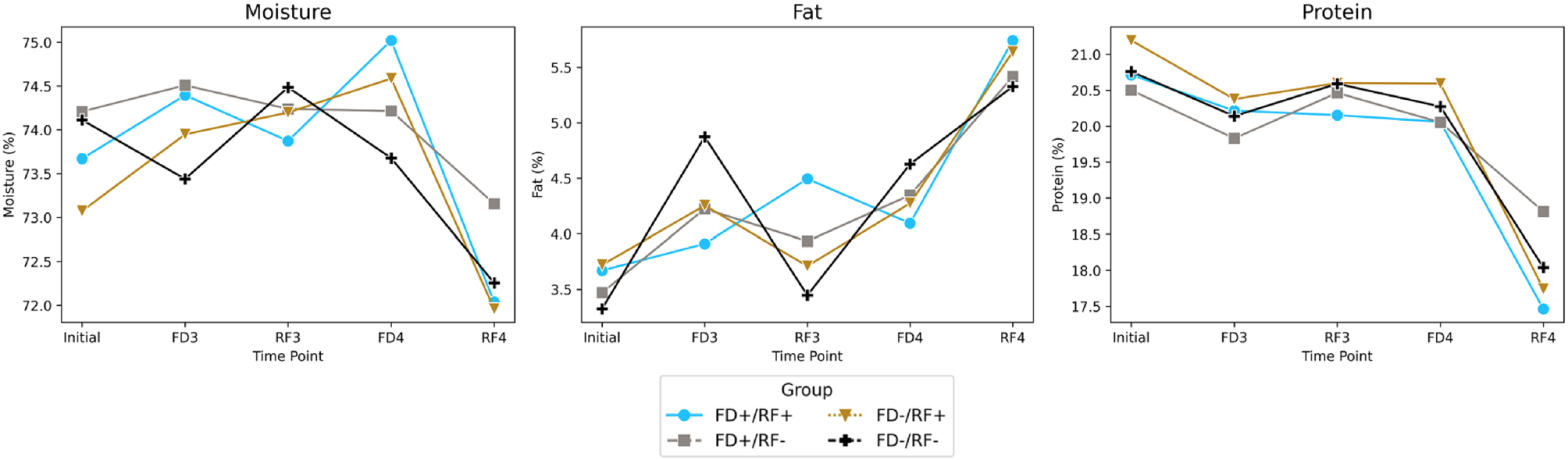
Moisture, protein and fat content of muscle during the second feeding challenge.

While FD and RF periods always had an effect on the proximate composition of the muscle, grouping only had a significant impact on moisture. Interaction of the two factors only had an effect on muscle protein content (Table 4). As a general pattern, fish tend to maintain lipid and protein content during the feeding challenge, however an opposite pattern of losing protein and retaining lipid was observed during RF4 (Fig. 6). This may be explained by fish age and photoperiod as they were approaching maturity during the fall of their second year of life.

**Table 4:** Proximate composition of muscle during the second feeding challenge. Nine fish per group per period were sampled.

Time points	Group	Lipid	Protein	Moisture	Ash	Energy
Initial	FD + /RF +	3.67 ± 0.21	20.71 ± 0.36	73.67 ± 0.81	5.64 ± 0.35	5622.67 ± 11.25
	FD + /RF-	3.47 ± 0.13	20.5 ± 0.44	74.21 ± 0.53	5.42 ± 0.15	5600.5 ± 40.15
	FD-/RF +	3.72 ± 0.62	21.19 ± 0.6	73.07 ± 1.22	5.46 ± 0.18	5602.0 ± 99.35
	FD-/RF-	3.32 ± 0.93	20.76 ± 0.28	74.11 ± 1.26	5.99 ± 0.45	5507.17 ± 232.23
FD3	FD + /RF +	3.91 ± 1.93	20.21 ± 0.11	74.4 ± 1.7	6.47 ± 0.86	5684.5 ± 245.57
	FD + /RF-	4.22 ± 2.13	19.83 ± 0.14	74.51 ± 1.92	6.50 ± 0.81	5750.5 ± 288.06
	FD-/RF +	4.25 ± 0.70	20.37 ± 0.15	73.95 ± 0.89	6.05 ± 0.79	5726.17 ± 78.89
	FD-/RF-	4.87 ± 1.85	20.14 ± 0.43	73.44 ± 2.56	6.15 ± 0.88	5792.34 ± 185.63
RF3	FD + /RF +	4.49 ± 0.60	20.15 ± 0.36	73.87 ± 0.75	6.73 ± 0.06	5749.0 ± 68.01
	FD + /RF-	3.93 ± 0.86	20.46 ± 0.27	74.24 ± 0.72	6.54 ± 0.22	5676.5 ± 132.94
	FD-/RF +	3.71 ± 0.23	20.6 ± 0.082	74.2 ± 0.18	6.94 ± 0.18	5641.0 ± 10.04
	FD-/RF-	3.45 ± 0.67	20.59 ± 0.21	74.49 ± 0.46	6.69 ± 0.63	5613.67 ± 78.62
FD4	FD + /RF +	4.09 ± 0.86	20.06 ± 0.81	75.02 ± 1.12	7.06 ± 0.55	5696.34 ± 169.28
	FD + /RF-	4.35 ± 0.94	20.05 ± 0.55	74.21 ± 0.89	7.42 ± 0.43	5735.34 ± 144.48
	FD-/RF +	4.27 ± 0.7	20.59 ± 0.31	74.59 ± 1.56	6.92 ± 0.3	5721.34 ± 135.01
	FD-/RF-	4.63 ± 0.35	20.27 ± 0.96	73.68 ± 0.46	6.97 ± 0.55	5794.0 ± 57.25
RF4	FD + /RF +	5.74 ± 0.79	17.46 ± 0.53	72.04 ± 0.85	6.25 ± 0.49	5935.67 ± 73.12
	FD + /RF-	5.41 ± 0.23	18.81 ± 0.53	73.16 ± 0.36	6.26 ± 0.35	5862.34 ± 25.98
	FD-/RF +	5.64 ± 0.26	17.74 ± 0.84	71.96 ± 0.45	6.23 ± 0.32	5840.5 ± 64.21
	FD-/RF-	5.32 ± 0.47	18.04 ± 0.44	72.26 ± 0.41	6.5 ± 0.38	5812.0 ± 68.52
*p*-Value (period)		4.93E–04***	1.40E−16***	0.000493***	3.37E−07***	2.39E-04***
*p*-Value (group)		0.584163	0.2119875	5.84E−01***	0.890414	0.886895584
*p*-Value (period × group)		0.869013	1.74E−01***	0.8690129	0.862933	0.921653729

***The p value less than 0.05 are significantly different among the four groups.

After constituting the groups, muscle fatty acid composition was surveyed during the second feeding challenge (Table 5a and b). Feeding periods had a significant effect on all fatty acids except C18:1n-9 (Oleic acid, *P* = 0.095), C18:3n-3 (⍺-linolenic acid, *P* = 0.45), C20:2n–6 (Eicosadienoic acid, *P* = 0.6) and C20:3n–6 (Dihomo-γ-linolenic acid, *P* = 0.14) which remained consistent throughout the second challenge. However, fatty acid composition was not affected by grouping. Similarly, period and group interaction were found to have no effect on any of the fatty acids in the muscle. The overall variations of the major fatty acid categories are summarized in Table 5B. Among all four groups FD + /RF− was the only group to maintain a similar mono-unsaturated fatty acid (MUFA) content throughout the challenge while other groups began to accumulate MUFAs after the last FD period. Poly unsaturated fatty acids (PUFAs) and saturated fatty acids (SAT) showed an overall increase during the four steps of the second feeding challenge.

**Table 5:** Fatty acid composition (% of total lipid) of fillet of rainbow trout during the second feeding challenge.

Time points	Group	C14:0	C16:0	C18:0	ƩSAT	C16:1	C18:1n-9	C20:1n-9	ƩMUFA		
		Myristic acid	Palmitic acid	Stearic acid		Palmitoleic acid	Oleic acid	11-Eicosenoic acid or Gondoic acid			
(Part A)											
Initial	FD + /RF +	1.8278 ± 0.0005	21.0978 ± 0.0041	4.7469 ± 0.0021	64.0567 ± 0.0034	6.7998 ± 0.0006	29.5829 ± 0.002	2.4435 ± 0.0007	12.1367 ± 0.0044		
	FD + /RF-	1.7641 ± 0.0012	20.9589 ± 0.0006	4.5227 ± 0.0012	63.6167 ± 0.0119	6.5368 ± 0.0047	29.8354 ± 0.0057	2.5035 ± 0.0003	12.1967 ± 0.0009		
	FD−/RF +	1.8039 ± 0.0022	20.355 ± 0.0084	4.4847 ± 0.0013	63.3033 ± 0.0179	6.6375 ± 0.0068	30.0193 ± 0.0049	2.543 ± 0.0031	12.4467 ± 0.0049		
	FD−/RF−	1.6676 ± 0.0016	20.3468 ± 0.0052	4.5758 ± 0.0015	62.52 ± 0.0227	6.2086 ± 0.0074	29.7205 ± 0.008	2.7045 ± 0.003	12.4333 ± 0.0036		
FD3	FD + /RF +	1.5575 ± 0.0006	20.0387 ± 0.0056	4.3216 ± 0.001	62.1733 ± 0.0182	6.3213 ± 0.0073	29.9354 ± 0.0179	2.655 ± 0.0002	12.0967 ± 0.0067		
	FD + /RF−	1.5853 ± 0.0028	20.0121 ± 0.0073	4.5548 ± 0.0024	62.64 ± 0.0314	6.3038 ± 0.0117	30.1837 ± 0.0133	2.8049 ± 0.0029	12.1667 ± 0.0014		
	FD−/RF +	1.6141 ± 0.0009	19.7536 ± 0.0023	4.3777 ± 0.0013	62.5067 ± 0.0087	6.3497 ± 0.0043	30.4116 ± 0.0024	2.6569 ± 0.0016	12.56 ± 0.0026		
	FD−/RF−	1.6403 ± 0.0013	20.0445 ± 0.009	4.3634 ± 0.0025	63.1633 ± 0.0223	6.6461 ± 0.0071	30.4707 ± 0.0027	2.5501 ± 0.0017	12.3167 ± 0.0071		
RF3	FD + /RF +	1.6824 ± 0.0006	20.7828 ± 0.0024	4.4749 ± 0.0008	64.02 ± 0.0003	6.9248 ± 0.0014	30.1575 ± 0.0038	2.3442 ± 0.0014	12.1767 ± 0.0023		
	FD + /RF−	1.6119 ± 0.0015	20.3233 ± 0.0048	4.4961 ± 0.0017	62.6067 ± 0.0076	6.2883 ± 0.0028	29.8898 ± 0.0082	2.4546 ± 0.0026	12.2433 ± 0.0039		
	FD−/RF +	1.6366 ± 0.0001	20.4421 ± 0.004	4.1889 ± 0.0011	61.9233 ± 0.0067	6.1797 ± 0.002	29.4775 ± 0.0054	2.5977 ± 0.0012	12.5167 ± 0.005		
	FD−/RF−	1.6453 ± 0.0009	20.5796 ± 0.0034	4.4093 ± 0.0009	62.6667 ± 0.0045	6.4838 ± 0.0026	29.547 ± 0.0058	2.3315 ± 0.0012	12.2033 ± 0.0038		
FD4	FD + /RF +	1.6567 ± 0.0017	20.1115 ± 0.0021	4.4487 ± 0.0013	62.8733 ± 0.0112	6.6561 ± 0.0048	30.0036 ± 0.0051	2.3654 ± 0.0024	13.1533 ± 0.0146		
	FD + /RF−	1.551 ± 0.0007	20.3422 ± 0.0025	4.4039 ± 0.0008	63.1667 ± 0.0027	6.6835 ± 0.0035	30.1842 ± 0.0023	2.2371 ± 0.0002	11.9667 ± 0.0002		
	FD−/RF +	1.6373 ± 0.0015	20.7404 ± 0.006	4.4657 ± 0.0026	61.77 ± 0.0317	6.419 ± 0.0082	28.5057 ± 0.0308	2.4202 ± 0.0023	12.9133 ± 0.0073		
	FD−/RF−	1.6769 ± 0.0006	19.7024 ± 0.0025	4.3694 ± 0.0018	63.0433 ± 0.0086	6.8509 ± 0.0026	30.4411 ± 0.0096	2.5013 ± 0.0012	13.29 ± 0.0084		
RF4	FD + /RF +	1.8273 ± 0.0011	20.3163 ± 0.0042	4.5457 ± 0.0007	64.9933 ± 0.0037	7.5477 ± 0.0049	30.7573 ± 0.0044	2.4404 ± 0.0012	13.2833 ± 0.0145		
	FD + /RF−	1.6957 ± 0.0007	21.0809 ± 0.002	4.4498 ± 0.0006	65.4167 ± 0.0115	7.1762 ± 0.0028	31.0182 ± 0.0068	2.3773 ± 0.0011	12.12 ± 0.0013		
	FD−/RF +	1.5744 ± 0.0018	20.3452 ± 0.012	4.3435 ± 0.0028	63.9167 ± 0.0383	6.8197 ± 0.0055	30.8316 ± 0.0167	2.5071 ± 0.0023	12.1933 ± 0.0073		
	FD−/RF−	1.7205 ± 0.0002	20.6855 ± 0.0037	4.3482 ± 0.0011	64.2667 ± 0.0071	6.9989 ± 0.0024	30.5132 ± 0.0053	2.4374 ± 0.0008	12.7667 ± 0.0062		
*P*-value (Time point)		0.0265231*	0.0098728**	0.0377131*	0.03304733517*	0.0151821*	0.095079459	0.0031691***	0.174451829		
*P*-value (group)		0.506083942	0.489478369	0.0936461	0.448398197	0.312895794	0.79230104	0.523703357	0.19903086		
*P*-value (Time point × group)		0.723457976	0.326836156	0.455290928	0.974680216	0.879843959	0.840963884	0.441030565	0.561316147		

Time points	Group	C18:2n-6	C18:3n-3	C20:2n-6	C20:3n-6	C20:4n-6	C20:3n-3	C20:5n-3	C22:5n-3	C22:6n-3	ƩPUFA
		linoleic acid	α-Linolenic acid	Eicosadienoic acid	Dihomo-γ-linolenic acid	Arachidonic acid	Eicosatrienoic acid	Eicosapentaenoic acid	Docosapentaenoic acid	Docosahexaenoic acid	
(Part B)											
Initial	FD + /RF +	8.5749 ± 0.0029	1.0236 ± 0.0008	0.3489 ± 0.0005	0.2074 ± 0.0001	0.7858 ± 0.0003	0.1774 ± 0.0002	7.5572 ± 0.0016	1.9054 ± 0.0012	12.65 ± 0.0057	23.63 ± 0.0052
	FD + /RF−	8.626 ± 0.0012	1.0192 ± 0.0004	0.354 ± 0.0001	0.2136 ± 0.0002	0.8274 ± 0.0006	0.1927 ± 0.0002	7.8135 ± 0.0038	2.0549 ± 0.0019	12.5568 ± 0.0065	24.0133 ± 0.0125
	FD−/RF +	8.802 ± 0.0018	1.0099 ± 0.0004	0.3646 ± 0.0002	0.2149 ± 0.0002	0.7838 ± 0.0016	0.1632 ± 0.0007	7.7077 ± 0.0055	2.0128 ± 0.0003	12.7543 ± 0.0045	24.0033 ± 0.0124
	FD−/RF−	8.6328 ± 0.0009	1.0488 ± 0.0005	0.3852 ± 0.0002	0.2064 ± 0.0001	0.8241 ± 0.0014	0.202 ± 0.0008	7.9815 ± 0.0072	2.0132 ± 0.0014	13.2769 ± 0.011	24.89 ± 0.0211
FD3	FD + /RF +	8.4907 ± 0.0063	0.9495 ± 0.0004	0.3764 ± 0.0004	0.2256 ± 0.0001	0.8326 ± 0.0007	0.185 ± 0.0002	7.5267 ± 0.0043	2.0581 ± 0.0003	14.493 ± 0.021	25.6967 ± 0.0248
	FD + /RF−	8.4015 ± 0.0029	0.9599 ± 0.0003	0.3667 ± 0.0005	0.198 ± 0.0004	0.8808 ± 0.0018	0.2109 ± 0.0006	7.6432 ± 0.0044	2.0611 ± 0.0009	13.7732 ± 0.0225	25.1333 ± 0.031
	FD−/RF +	8.907 ± 0.0011	0.9967 ± 0.0004	0.3708 ± 0.0003	0.2115 ± 0.0002	0.8489 ± 0.0009	0.231 ± 0.0004	7.2477 ± 0.0027	2.08 ± 0.0008	13.8591 ± 0.0083	24.85 ± 0.0061
	FD−/RF−	8.7905 ± 0.0049	0.975 ± 0.0006	0.3711 ± 0.0002	0.2189 ± 0.0001	0.8159 ± 0.0016	0.2086 ± 0.0009	7.4789 ± 0.0041	2.0194 ± 0.0021	13.3158 ± 0.0079	24.43 ± 0.0153
RF3	FD + /RF +	8.8042 ± 0.0018	1.028 ± 0.0014	0.3375 ± 0.0001	0.1973 ± 0.0	0.7028 ± 0.0001	0.1694 ± 0.0001	7.3327 ± 0.0024	2.1135 ± 0.001	12.9066 ± 0.0007	23.7567 ± 0.0021
	FD + /RF−	8.7977 ± 0.0029	0.9887 ± 0.0004	0.3606 ± 0.0003	0.186 ± 0.0002	0.7392 ± 0.0004	0.149 ± 0.0002	7.3646 ± 0.0011	2.2102 ± 0.0008	14.1177 ± 0.0097	25.1267 ± 0.0106
	FD−/RF +	8.9079 ± 0.0036	1.0107 ± 0.0004	0.3715 ± 0.0002	0.2193 ± 0.0003	0.8296 ± 0.0003	0.1679 ± 0.0003	7.5521 ± 0.002	2.1518 ± 0.0016	14.2182 ± 0.0079	25.5133 ± 0.0099
	FD−/RF−	8.8973 ± 0.0031	0.9748 ± 0.0005	0.3538 ± 0.0003	0.1856 ± 0.0001	0.7491 ± 0.0002	0.1734 ± 0.0	7.3259 ± 0.0036	2.1809 ± 0.0015	14.1412 ± 0.0119	25.11 ± 0.0081
FD4	FD + /RF +	9.7278 ± 0.0111	1.0607 ± 0.002	0.3565 ± 0.0005	0.2058 ± 0.0002	0.7272 ± 0.0011	0.1517 ± 0.0005	6.7742 ± 0.0074	2.3004 ± 0.0025	13.4223 ± 0.0154	23.9367 ± 0.0256
	FD + /RF−	8.806 ± 0.0004	0.9228 ± 0.0001	0.3312 ± 0.0002	0.1908 ± 0.0002	0.7189 ± 0.0003	0.1437 ± 0.0001	7.3174 ± 0.0017	2.2923 ± 0.0007	13.7988 ± 0.004	24.7967 ± 0.0029
	FD−/RF +	9.4863 ± 0.0051	1.0072 ± 0.0006	0.3703 ± 0.0003	0.2037 ± 0.0003	0.7726 ± 0.0012	0.1582 ± 0.0002	7.2705 ± 0.0079	2.363 ± 0.0012	14.0919 ± 0.0168	25.23 ± 0.0276
	FD−/RF−	9.6922 ± 0.0074	1.0952 ± 0.0019	0.3732 ± 0.0005	0.1912 ± 0.0003	0.7021 ± 0.0003	0.1377 ± 0.0001	6.9443 ± 0.0049	2.2891 ± 0.0009	12.9846 ± 0.0058	23.6233 ± 0.0031
RF4	FD + /RF +	9.6812 ± 0.0103	1.1603 ± 0.0031	0.3805 ± 0.0002	0.214 ± 0.0003	0.6421 ± 0.0001	0.1638 ± 0.0002	6.4907 ± 0.0068	2.1301 ± 0.0027	11.5534 ± 0.0063	21.5733 ± 0.0128
	FD + /RF−	8.7725 ± 0.0019	0.9675 ± 0.0002	0.3257 ± 0.0001	0.1727 ± 0.0001	0.682 ± 0.0003	0.1659 ± 0.0002	7.0025 ± 0.0032	2.0413 ± 0.0012	11.9789 ± 0.0069	22.37 ± 0.0108
	FD−/RF +	8.6817 ± 0.0062	1.0052 ± 0.0013	0.3479 ± 0.0004	0.1971 ± 0.0003	0.6247 ± 0.0003	0.0812 ± 0.0008	7.0216 ± 0.0067	2.1747 ± 0.0023	13.3913 ± 0.0305	23.84 ± 0.0395
	FD−/RF−	9.241 ± 0.0043	1.0874 ± 0.0012	0.3545 ± 0.0002	0.2041 ± 0.0002	0.6931 ± 0.0005	0.1566 ± 0.0003	6.7701 ± 0.002	2.048 ± 0.0005	12.7037 ± 0.0046	22.93 ± 0.006
*p*-value (Time point)		0.0015647***	0.449740864	0.607877716	0.135670645	0.0001178***	0.0029413**	0.0001542***	0.000064***	0.02178734*	0.01798366092*
*p*-value (group)		0.144909572	0.28838482	0.31671723	0.079557304	0.713313444	0.789716276	0.372217184	0.737278478	0.561940556	0.521070269
*p*-value (Time point × group)		0.504056685	0.885364453	0.700632472	0.683441163	0.921131768	0.545994749	0.945130189	0.983744936	0.841649354	0.935568509

*, **, ***The p value less than 0.05 are significantly different among the four groups.

## Discussion

Consumption of sustainable feed sources such as plant-based protein plays a major role in long-term aquaculture sustainability. Although the rainbow trout strain that we used in this study has been selected for high growth on plant-based diet for eight generations and has always been reared in the same environment, our results showed that inter-individual variations in response to FD and RF as well as feed efficiency are still present in the population. This has been evidenced by the significant differences in weight gain and TGC in the present study. It has been suggested that an average of 12% genetic gain is produced through selective breeding per generation in terms of growth performance^24^. As previously mentioned by Overturf et al.^27^, selection could improve tolerance to antinutritional factors, palatability sense, metabolic regulations and feed intake. Variations in fish strain play a key role in responding to the compound diets as various independent or interactive physiological changes take place.

In this research we aimed to examine the indirect criteria for improvement of feed utilization efficiency to be used in future rainbow trout breeding programs to address the sustainability issue from a different angle. Based on the previous research, we hypothesized that fish’s response to consecutive periods of FD and RF during compensatory feeding regime has a positive correlation with feed utilization efficiency^13,15,20,22^. Research has shown a close association between FD and changes in digestive enzyme activities, gut microbial communities, metabolic pathways, and immune responses in fish^30–32^. All these changes are linked to the digestive and utilization performance of fish which will consequently affect FCR. Differences in weight gain between the FD + and FD− groups appeared after the 1^st^ FD with the FD + groups remaining the lower weight groups for the rest of the study. Such observation could imply that the fish who lost more weight in early stages remained smaller throughout the compensatory feeding. Therefore, using such benchmark might enable the researchers for an early detection of efficient fish, although more research is required. The current study presents evidence that shows the measured indices can be used as an indirect selection criterion for FE because not only is it easy to record under any rearing condition but also the observed variations are correlated with FCR as a measure of feed utilization efficiency. With repetition of the indirect criteria, we have shown that some fish demonstrate consistent behavior throughout time and life stages. Selection of such fish can guarantee the improvement of feed utilization efficiency in a breeding plan. In a recent study by De Verdal et al.^33^, a direct selecting breeding program using videorecording was developed to improve FCR in juvenile Nile tilapia, *Oreochromis niloticus*. These authors indicated that a 12% difference in FCR between two divergent lines of Nile tilapia was observed with a 4% improvement of phenotypic FCR per generation. This is almost in line with our results as the FCR for FD−/RF + was significantly lower compared to FD−/RF− and FD + /RF− groups. In another study, the FCR of seabass was improved within a selective breeding program with feed intake measurement of 588 individuals^34^. In Finland, within a national selective breeding program for rainbow trout, FCR was improved by 11.6% after eight generations^35^. These authors reported that genetic improvements of rainbow trout stocks significantly contribute to lowering feed costs (18.3%), production costs (7.8%) and disposal of phosphorus and nitrogen (18.3%). Improved FCR could be considered a valuable trait, especially with zero fishmeal diets, which can potentially have significant economic and environmental impacts in trout aquaculture. Therefore, inclusion of individual FCR in selective breeding programs is a practical approach towards aquaculture sustainability.

Our results for proximate composition of muscle during the second feeding challenge did not reflect those observations for growth and FCR. Long periods of feed deprivation did not affect the muscle lipid and protein contents. Generally, protein deposition is dependent on feed protein content, amino acid composition of dietary protein, non-protein energy intake and the protein-sparing effect of lipid in diet. Lipids will help to conserve the existing protein exclusively for growth purposes while only negligible protein catabolism occurs in the animal body36. Our selected trout was resistant to losing the nutrient (protein and lipid) content of muscle during the feeding challenge regardless of feed deprivation and refeeding except at the end of feed deprivation challenge during maturation period. In general, standard metabolic rate (SMR) is influenced by body mass, with large fish using more oxygen per unit time, but less oxygen per unit mass per unit time. While we speculate that under restricted feeding fish utilized the visceral fat to cover their basal metabolism, our study found that extended periods of feed deprivation did not affect the lipid and protein contents of muscle during the second feeding challenge. Different fish species, sizes and strains have shown different tolerance to FD periods^15^ as is proven in our findings. Current study presents evidence for variation of response to FD and RF periods in terms of weight gain and loss among selected line of rainbow trout. Such results along with the estimated heritability for different measures of feed efficiency ranging from 0.03 to 0.23^11,20^, suggests that selection based on such criteria can improve feed efficiency indirectly without negative effect on nutritional values of muscle. However, to understand the basics of energy supply and lipid turnover further investigation has to be done. While lipid content is comparable between groups, the fatty acid composition of muscle was significantly different between FD and RF periods during the second challenge. α-Linolenic acid, which is the precursor for the omega-3 fatty acid biosynthesis pathway, remained unchanged through all the starvation and refeeding periods. Similarly, Eicosadienoic acid and Dihomo-γ-linolenic acid both of which possess a higher position in the omega-6 fatty acid biosynthesis pathway remained consistent.

The main question in this study was whether body weight variation during FD and RF had a relationship with feed efficiency and this was observed as the group with lowest weight loss during FD and highest weight gain during RF had the lowest FCR among all. Since the growth rate stabilizes as the fish ages^37,38^, growth differences were continually observed among the individuals within the population in terms of body weight gain per day during the second year of this study. In the Compensatory feeding regimen, this was only true until the fish got into the maturation phase and significant differences were observed in BWG in RF4.

Through repeated assessments of this criterion, some fish have been identified as consistently efficient feed users throughout their lifetimes, making them ideal candidates for selection in breeding programs to improve the feed efficiency. Improving feed efficiency is a valuable achievement, particularly with zero fishmeal diets, as it can have significant economic and environmental benefits for trout aquaculture. Thus, incorporating individual FCR into selective breeding programs is a practical approach to promoting sustainability in aquaculture. To confirm the results of such selection criteria to improve feed efficiency, evaluation of the second generation is proposed as the future direction of this study.

## Conclusions

In conclusion, our study demonstrated a clear correlation between weight loss during four-week feed deprivation periods and weight gain during a subsequent four-week periods of re-feeding with variations in FCR in rainbow trout. These findings suggest that these traits could potentially serve as indirect indicators for improving FCR through selective breeding, assuming that they are heritable. Our results highlight that selecting FD-/RF + fish with the lowest FCR (0.99), can improve feed efficiency without affecting the fillet nutritional values. The combination of FD and RF as indirect criteria holds great potential for selecting individuals with reduced FCR. However, in order to determine the potential genetic gain that a breeding program based on these traits could generate in terms of FCR reduction, as well as reduced feed costs and effluents in rainbow trout culture, it is necessary to estimate their heritability and genetic correlation with FCR.

## Data Availability

The datasets used and/or analyzed during the current study are available from the corresponding author on reasonable request.
